# Mixed methods, mixed outcomes? Combining an RCT and case studies to research the impact of a training programme for primary school science teachers

**DOI:** 10.1080/09500693.2018.1563729

**Published:** 2019-01-07

**Authors:** Judith Bennett, Pam Hanley, Ian Abrahams, Louise Elliott, Maria Turkenburg-van Diepen

**Affiliations:** a Department of Education, University of York, York, UK; b School of Education and Professional Development, University of Huddersfield, Huddersfield, UK; c School of Education, University of Lincoln, Lincoln, UK; d York Trials Unit, University of York, York, UK

**Keywords:** Mixed methods, randomised controlled trial, teacher professional development

## Abstract

A randomised controlled trial (RCT) and a series of case studies were used to determine the impact of two variants of an intervention (a professional development programme) aimed at improving primary school science teachers’ subject and pedagogic content knowledge, and enhancing their subject leadership ability. Ninety-six schools were randomly assigned to full or partial treatment groups or a ‘business-as-usual’ control group. Quantitative data were collected from teachers and pupils through an assessment of scientific knowledge based on standardised assessment items. Qualitative data were collected through interviews and lesson observation initially in thirty case study schools. There were three data collection points: pre- and post-intervention, and one year later.

[Guskey, T. (1986). Staff development and the process of teacher change. *Educational Researcher*, *15*(5), 5–12.] *Levels of Professional Development Evaluation* model was used as the analysis framework. The quantitative data from the teachers’ subject knowledge assessment indicated neither the full nor the partial training programmes had a statistically significant impact on teachers’ performance. In contrast, the qualitative data suggested that many teachers in the full treatment group believed that their subject knowledge had improved and reported increased confidence in their teaching of science. Lesson observations provided corroborating evidence of change in teachers’ practice, and some modest evidence of wider change in schools. There was no statistically significant improvement in pupil performance in subject knowledge assessments when teachers had participated in the intervention. In the context of research methods, the study suggests that a mixed-methods approach to evaluation is likely to yield a more rounded and nuanced picture of the overall impact of an intervention.

## Introduction, background and study aims

In primary schools in England, attended by pupils aged 5–11, much of the science teaching is undertaken by teachers with only basic qualifications in science. One outcome of this has been the creation of Continuing Professional Development (CPD) programmes to support such teachers in their teaching of science. This study reports on the impact of one such programme, designed to equip teachers responsible for teaching science in primary schools with subject-specific knowledge and pedagogical content knowledge (PCK), together with leadership training, so that they could lead the science provision in their school. The programme was specifically aimed at primary school teachers who were designated as ‘science specialists’ within their schools but who had had no formal science qualifications beyond the age of 16 and who also did not have any initial teacher training qualification specifically relating to science.

The principal aim of this evaluative study was to provide a high-quality evidence base of school, teacher and pupil-level impacts of the training programme, immediately after completion and one year later, to inform judgements about a possible national roll-out.

Within the broad overall aim, the study was designed to address the following research questions:

What impact did the Continuing Professional Development (CPD) programme have on:

- science subject knowledge and science pedagogic content knowledge (PCK) of participating teachers?
- the science subject knowledge and science PCK of teacher colleagues of participating teachers, (i.e. impact in school)?
- teachers’ confidence in their science teaching?
- the science subject knowledge of pupils taught by participating teachers?

What teacher-level and school-level factors affected engagement with the programmes?

What was the impact of CPD ‘dose’, (i.e. comparing impacts of longer and shorter versions of the CPD)?

The paper draws on work undertaken for a large-scale project reported elsewhere (Abrahams et al., 2014).

## Context: approaches to educational evaluation

### Randomised controlled trials (RCTs)

The Randomised Controlled Trial (RCT) takes the form of a classic experimental design with subjects randomly assigned to either a control or an intervention group. Oakley (2000) describes RCTs as follows:

An RCT is simply an experiment (‘trial’) which tests alternative ways of handling a situation. Sometimes the intervention is tested against what would have happened had it not been used; sometimes different interventions are compared. (p. 18)

RCTs are widely used in clinical trials and advocacy of their more widespread use in education research dates back to the second half of the 1990s (Hargreaves, 1996), during a period of intense debate, both nationally and internationally, over the nature and purpose of educational research, and the extent to which it contributes – or fails to contribute – to the effectiveness of educational provision (e.g. Hillage, Pearson, Anderson, & Tamkin, 1998, in the UK; Shavelson & Towne, 2001, in the USA). However, their use has not been without controversy. For some (e.g. Oakley, 2000; Torgerson & Torgerson, 2001), they are a means of providing hard evidence on the effectiveness of educational interventions and have been promoted as the ‘gold standard’ methodology for answering the question, *what works?* For others (e.g. Hammersley, 2001) the degree of complexity of educational contexts requires insights and explanations that draw on a wider range of strategies, and RCTs are seen as largely inappropriate.

Whilst there has been an increase in the use of RCTs in educational research, there has also been a move towards mixed-methods research (see, for example, Gorard & Taylor, 2004), which involves gathering, analysing, and integrating data from both quantitative and qualitative research in a single study. Underpinning mixed-methods approaches is the notion that a combination of approaches provides a broader understanding of a research problem than either approach used in isolation, thus enhancing the validity of the findings (see, for example, Hanley, Chambers, & Haslam, 2016).

### Assessing the impact of an intervention

Several models have been proposed for assessing the impact of an intervention. They are characterised by two emphases: one on the process of bringing about change in teachers’ practices and the other on outcomes. In an early and influential publication, Fullan (1982) proposed three possible dimensions to implementing a new programme: the use of new or revised materials, the use of new teaching approaches, and the alteration of beliefs. Work in the 1980s and 1990s placed a strong emphasis on staff development through CPD, involving the provision of resources to use in lessons, practice in using the resources, and giving teachers on-the-job feedback (e.g. Harland & Kinder, 1997; Joyce & Showers, 1980, 1995; Kinder & Harland, 1991; Kinder, Harland, & Wootten, 1991).

The last two decades have been characterised by an intensifying climate of accountability, and consequently the focus of the impact of CPD has shifted towards outcomes, particularly those relating to pupil learning and performance. A widely-used means of identifying different outcomes is Guskey’s *Levels of Professional Development Evaluation Model* (Guskey, 1986, 2000), which identifies five levels of impact:

1. Participants’ reactions to the programme provision
2. Participants’ learning
3. Organisational (i.e. school or department) support and change
4. Participants’ use of new knowledge and skills
5. Pupil learning outcomes

Guskey sees CPD impact as linear progression through these levels, i.e. impact at one level is only possible if impact has taken place at previous levels. In contrast to earlier models, Guskey’s focus is the outcomes of the CPD episode, not its process or content, with the highest level of desirable impact being at level 5, impact on pupils’ learning.

Muijs and Lindsay (2008) surveyed a number of evaluations and found that they most frequently focused on Level 1 evaluations (participants’ responses) followed by Level 2 evaluations (participants’ learning). They also found that Level 5 evaluations (impact on pupils) were more common than Level 3 and 4 evaluations (organisational change and use of new knowledge and skills), pointing to a higher priority being given to test results as success indicators.

The emphasis of the study reported here was on impacts on teachers (science subject knowledge and pedagogic content knowledge), impacts on colleagues in teachers’ schools, and impacts on pupils’ learning. Guskey’s model therefore lent itself well to providing the theoretical framework for the analysis and discussion of the data.

### Primary teacher continuing professional development (CPD)

A systematic review of elementary science programmes by Slavin, Lake, Hanley, and Thurston (2014) found a small number of experimental evaluations that used randomised or matched control groups. The review found that approaches emphasising professional development had the most impact on pupil academic achievement, achieving at least moderate effect sizes of 0.20 or more.

One of the studies identified as effective by Slavin et al. (2014) centred on increasing conceptual challenge (Mant, Wilson, & Coates, 2007). The approach later evolved into the Thinking, Doing, Talking Science (TDTS) programme comprising a series of professional development sessions, resources and support for primary school teachers. The aim was to improve pupils’ thinking skills and science attainment by making science lessons more conceptually challenging, more practical, and more interactive. An RCT using the same pupil attainment measure as the study reported in this article found the intervention group scored higher than the control pupils (effect size of 0.22), with a greater effect apparent for girls than for boys (Hanley, Slavin, & Elliott, 2015).

## The CPD programme

The CPD programme had three principal components: subject knowledge, pedagogical content knowledge and subject leadership. Two versions of the programme were offered to explore the effects of ‘dose’. The full version addressed all three strands and comprised fourteen days of training in two-and three-day residential blocks during one school year, together with ten further days of support through network meetings, online provision and dedicated time in school. The shorter version comprised two two-day blocks and focused on the pedagogic content knowledge and school leadership strands. Subject knowledge components included extending knowledge of the *Big Ideas* (Harlen, 2010) in science, common misconceptions, and progression of subject knowledge to levels beyond that taught at primary schools. *Pedagogical Content Knowledge components focused on* current research and thinking about how children learn in science, curriculum design, assessment, and the use of digital technologies to enhance teaching. Subject Leadership components included understanding of the role of the Primary Science Specialist, challenges facing science subject leaders, auditing staff needs, mentoring and coaching colleagues and planning for school impact. Residential blocks were complemented by requirements to plan, implement and evaluate action plans for impact on practice.

## The design of the study

The two-year study adopted a mixed-methods approach, consisting of an RCT and a series of case studies. In the context of the drive to make more use of experimental techniques in educational research, a mixed methods approach conferred several benefits, permitting quantitative data from an RCT to be gathered to answer the question, *what works?* (or what does not work?), and qualitative data from case studies to be used to yield information on factors which may have influenced impacts.

The RCT comprised three groups: a *full treatment group*, where participating teachers experienced the full CPD programme, a *partial treatment group*, where participating teachers experienced the short CPD programme, and a control group, where the training programme was not provided. The design was a ‘delayed treatment-control group’ design as schools in the control group could opt to take the training programme after two years.

The case studies were conducted on a subset of approximately one-third of the schools in each of the three groups in the RCT.

### The sample

To be eligible for the study, schools were required to have a science specialist who taught pupils aged 7–10, and who had no formal science qualifications beyond age 16 and no science-specific initial teacher training qualification. Ninety-six schools were recruited to the study, located in three geographical areas in England. The sample contained a mix of inner city, urban, sub-urban and rural schools.

The schools were grouped into matching triplets based on attainment, school socio-economic context (using provision of free school meals to pupils as a proxy) and year group being taught science by the specialist. Attainment was based on the mean percentage of pupils achieving Level 4 (the expected standard for children at this age) and above on national Standard Assessment Tests (SATs) in English and mathematics over the previous three years. One school from each triplet was then randomly assigned to each of three groups, full intervention, partial intervention and control, meaning that each treatment group comprised 32 schools.

Data were collected at three points across the two years of the study. Some attrition of the sample occurred due to staff changes, staff illness, concerns about the time demands associated with the CPD programme, and concerns about being in the control group. Of the original 96 schools, 84 provided data in the second data collection stage, and 76 at the third stage, resulting in an overall attrition rate of 21%. This is comparable to that of other RCTs.^1^ These 76 schools form the basis of the analysis presented here. While it could be argued that only using schools that had provided data reliably at all three points might introduce some bias into the findings, it was felt that this potential drawback was outweighed by the robustness of the data provided and the facility to track individual teachers and pupils.

School participation is summarised in Table 1.

**Table 1.** Summary of school participation in the study.

Allocation (*N* = 96)	Allocated to full CPD group (*N* = 32)	Allocated to partial CPD group (*N* = 32)	Allocated to control group (*N* = 32)
	Withdrew immediately after allocation (*N* = 6)	Withdrew immediately after allocation (*N* = 1)	Withdrew immediately after allocation (*N* = 4)
Received Baseline assessments (*N* = 85)	*N* = 26	*N* = 31	*N* = 28
	Discontinued intervention (*N* = 2)^a^	Discontinued intervention (*N* = 1)^a^	Withdrew (*N* = 1)
Received Post-intervention assessments (*N* = 84)	*N* = 26	*N* = 31	*N* = 27
	Insufficient data to continue (*N* = 1) No response (*N* = 2)	Insufficient data to continue (*N* = 1)	
Received One-year follow-up assessments (*N* = 80)	*N* = 23	*N* = 30	*N* = 27

^a^ Included in analysis as Intention To Treat (ITT).

To maximise retention, the study design followed recommendations for best practice, for example using a wait-list (i.e. all control schools were promised the treatment, or its equivalent, after the study was finished), and offering a financial incentive to the science specialist for returning data at each collection point.

### Data sources

The primary outcome measures for the RCT were (a) assessments of teacher subject knowledge completed by science specialists and nominated teaching colleagues, and (b) assessments of pupil subject knowledge. Data were collected at three points over the two-year duration of the study, i.e. pre-intervention (baseline), immediately after the intervention (post-test 1) and one year later (post-test 2).

The instruments used to assess teachers’ subject knowledge were past national SATs devised for 14-year-olds in England. These questions were used because they had already been subjected to reliability and validity tests. The questions in the instruments covered the full range of subject knowledge taught on the 11–14 curriculum as the coverage of the ‘Big ideas’ in science in the full CPD meant that teachers were exposed to most areas of the curriculum though the focus was on more difficult ideas. A different paper was administered at each data collection point to prevent teachers predicting questions and rehearsing the answers.

Pupils’ subject knowledge was assessed via instruments from a bank of standardised assessment items.^2^,^3^ Instruments were tailored to each year group, with five of the twenty items on each instrument overlapping with the paper for the year below and five with the paper for the year above. Teachers were asked to administer the pupil assessments under normal test conditions.

Teacher subject knowledge assessments were sent out to science specialists and to a teacher colleague nominated by the head teacher. Equivalent assessments were also administered six months later, and then a further twelve months later. Pupil data were collected at the same points.

Thirty case study schools, equally spread between the three groups, were identified on the basis of returning a full set of baseline data. Schools were visited twice during the project, shortly after teachers started the CPD intervention, and around one year later. Five schools were unable to participate in the second visit, making the final sample twenty-five schools. The visits had three main purposes: to conduct interviews with the science specialists about their beliefs and practices and, if relevant, their perceptions of the CPD; to gather the perspectives of other staff (the nominated colleague, and a member of the school senior management team); and to observe the specialist and their colleague teaching a science lesson.

Interviews with senior management staff were included to provide an additional perspective on outcomes reported by teachers, and to establish the nature of organisational support available to the teachers, a key determinant of success in Guskey’s work on the impact of CPD (1986, 2000). Teacher colleague interviews provided a perspective on how any impact on science leadership filtered through to those being led. The interviews with participants in the CPD focused on appropriateness of science knowledge tests (for them and their pupils), their views of the CPD and its impact on their scientific knowledge, confidence in teaching science, classroom practice, enactment of their role of science specialist, and science in their school more generally. They were also asked about support for science from their school’s senior management, about any other science-related CPD they had attended, and their views on the experience of being involved in an RCT. Teaching colleagues were asked about the impact of their science subject leader attending the CPD, on science subject leadership in their school, on their own science knowledge, on their classroom practice, on pupils and in the school more widely. Senior school managers were asked about the status of science in their schools, the support the school provided for science, and the impacts of their science subject specialist attending the CPD. The science specialists and teaching colleagues were also asked about aspects of their lesson that had been observed. In all cases, interviewees were probed for evidence to support what they said. All interviews were semi structured and audio- recorded.

Table 2 summarises the numbers of interviews conducted in each group of schools in the second phase of visits.

**Table 2.** Number of individuals who were interviewed in second phase of case study visits.

Participants	Full CPD Group	Partial CPD Group	Control
Science Specialists	8	7	7
Teaching Colleague	9	5	8
Senior Manager	7	6	6
Total interviews	24	18	21
Total schools	9	7	9

Lesson observations were undertaken to provide an additional source of evidence of potential impact on practice. Field notes were taken as records of the observations. To ensure consistency, moderating co-observations were undertaken.

Table 3 shows Guskey’s five levels and the associated methods of data collection.

**Table 3.** Guskey’s five levels and the associated methods of data collection used in the study.

Level	Focus of evaluation	Principal data collection method(s)
Impact at level 1	Participants’ reactions to the course provision	Interviews with Science Specialists
Impact at level 2	Participants’ learning	Science Specialists subject knowledge assessments Interviews with Science Specialists, Teaching Colleagues, School Management Team member
Impact at level 3	Organisational (i.e. school or department) change	Interviews with Science Specialists, Teaching Colleagues, School Management Team member
Impact at level 4	Participants’ use of new learning both in the class and in completing assessments	Field notes from lesson observations Interviews with Science Specialists, Teaching Colleagues, School Management Team member
Impact at level 5	Pupil learning and attitudinal outcomes^a^	Pupil subject knowledge assessments (and attitudinal surveys) Field notes from lesson observations Interviews with Science Specialists and Teaching Colleagues Pupil focus groups^b^

^a^ Attitudinal data is not reported in this paper.

^b^ Pupil focus groups are not reported in this paper.

### Data analysis

The teacher and pupil subject knowledge tests were marked using existing mark schemes. Total scores were calculated by summing the marks for each question. Interviews were transcribed and uploaded into qualitative data analysis software (NVivo) to facilitate exploring the data for patterns and trends. The initial round of thematic analysis was based on areas identified with reference to the interview schedules. Subtexts from the interview transcripts were selected and analysed under each theme. A second analysis focused on additional emerging themes. The cogency of these emerging themes was tested by matching them with evidence from the interview transcripts and field notes. A final set of themes was then selected for analysis.

More detailed statistical analysis was undertaken on the quantitative data:

- For teacher data, one-way between-groups analysis of covariance was used to look at the impact of the two variants of the CPD programme on the subject knowledge of the science specialists and their colleagues, using performance at baseline as the covariate.
- For pupil data, after adjusting for performance at baseline, test scores were subjected to multi-level modelling to account for pupils (level 1) being nested within schools (level 2). Factors such as year group and gender were included in the multi-level modelling analysis.

## Study findings

### Participants’ reactions to the programme provision

Participants’ reactions were gauged principally through the interviews with the science specialists, supplemented by information from interviews with their teaching colleagues and the school management team.

The science specialists were all positive about the CPD programme, with two main reasons being given. Firstly, the programme provided them with useful ideas and resources, including new and unfamiliar resources. For example:

I got lots of ideas about how to make [science lessons] more interactive … every now and then I find myself remembering things from the training and using them. … I hope that’s had an impact on my practice and it is more hands-on than it was maybe. (Science specialist, School 3, full intervention)

Secondly, the science specialists encountered activities and ideas that helped them in their role as subject leaders. Coaching of colleagues was one example valued by the science specialists:

There has been an emphasis on coaching, how to support colleagues in a one-to-one situation and to empower them to be confident in Science and the subject knowledge, so [I am] able to apply it and help my colleagues. (Science specialist, School 4, full intervention)

Four science specialists reported the science content that they were required to learn during the CPD was at a higher level than they had anticipated. For example:

Subject knowledge was, some of it was up there … that wasn’t just me that was lots of people saying that … (Science specialist, School 94, full intervention)

There were mixed views on the comparatively lower level of subject knowledge content in the partial intervention group. On balance, the science specialists felt happy with the amount, with some saying that it was material they could study in their own time if needed:

… I think that the knowledge side of it which seemed to be what the other group was offered, I could find out for myself, I felt. So I was more interested in the practical side of it. (Science specialist, School 85, partial intervention)

### Participants’ learning

Participants’ learning for the teachers in the two treatment groups was assessed primarily through the subject knowledge questionnaires. These consisted of questions from past national Standard Assessment Tests used with pupils at age 14 in England. They had a total of 150 marks, distributed as evenly as possible amongst the four areas of biology, chemistry, physics and scientific inquiry.

Tables 4 and 5 show the numbers of assessments completed and the scores obtained by the science specialist teachers and their teaching colleagues at the three points of data collection.

**Table 4.** Science specialist assessment performance.

		Baseline				Post-test 1				Post-test 2			
	No of assessments	Minimum	Maximum	Mean	SD	Minimum	Maximum	Mean	SD	Minimum	Maximum	Mean	SD
Full CPD group	16	56	113	84.3	20.3	46	124	87.3	23.7	71	120	96.8	17.4
Partial CPD group	19	26	129	90.8	25.0	33	144	95.9	27.5	63	134	99.5	27.6
Control group	16	61	127	92.3	19.8	61	120	94.3	15.5	73	121	95.1	12.9

**Table 5.** Teaching colleague assessment performance.

		Baseline				Post-test 1				Post-test 2			
	No of assessments	Minimum	Maximum	Mean	SD	Minimum	Maximum	Mean	SD	Minimum	Maximum	Mean	SD
Full CPD group	13	29	111	84.0	25.4	43	136	89.0	25.7	47	142	92.8	27.7
Partial CPD group	16	44	120	95.1	23.4	41	139	96.3	25.9	63	125	98.0	22.6
Control group	12	61	114	91.9	17.0	61	136	93.8	26.5	77	137	100.6	19.1

#### The baseline (pre-intervention) data

Assessments were returned by 68 of the 85 schools remaining after randomisation. The response rates for the full intervention group, partial intervention group and control groups were 67%, 80% and 80% respectively. The lower response rate for the full intervention group was a result of some assessments being discounted as they had been completed after teachers embarked on the CPD programme. Such teachers had therefore had opportunities to boost their subject knowledge, affecting the validity of the baseline data. The average mark achieved was 59%, although this covered a wide range from a maximum of 95% through to a minimum of 17%.

A series of analyses was conducted on the baseline data to assess the similarity of the three groups. The average marks were 56% (full CPD group), 61% (partial CPD group) and 57% (control group), with no statistically significant differences. Nor was there any statistically significant difference between the marks achieved by the science specialists (57%) and their teacher colleagues (60%).

#### The post-test 1 data

84 schools were still involved in the project at post-test 1. Of these, the schools who had provided pre-intervention data returned 51 specialist assessments and 41 colleague assessments at post-test 1.

One-way between-groups analyses of covariance was undertaken to look at the outcomes of the two CPD programmes on specialists’ and colleagues’ subject knowledge. The relevant group’s performance on a comparable assessment in the baseline data was used as the covariate in the analysis, enabling adjustment for pre-intervention scores when comparing post-intervention performances.

The specialists’ average marks (out of 150) at post-test 1 were 87.3 for the full CPD group (58%), 95.9 for the partial CPD group (64%) and 94.3 for the control group (63%) (see Table 4). There was no statistically significant difference in the change in scores between the different groups [*F*_2,47 _= 0.269, *p* = .765] from the baseline assessment to the post-test 1 assessment. As with the baseline data, the assessment results showed considerable differences in knowledge and understanding, with marks ranging from 33 to 144 out of a possible 150.

The colleagues’ average assessment scores were very similar to those of the specialists, with a similar spread of performance (from 41 to 139 at post-test). Again, there was no statistical difference in the scores from baseline to post-test 1 [*F*_2,37_ = 0.042, *p* = .959].

#### The post-test 2 data

Of the original 96 schools, 80 were still participating in the project at the point where the post-test 2 data were collected, comprising 23 in the full CPD group, 30 in the partial CPD group and 27 in the control group. Four failed to return any data in the third stage (three partial, one control) making the final sample 76 schools. The final sample used in the analysis comprised the 40 science specialists and 32 colleagues who had provided data at all three data collection points.

The specialists’ average marks at post-test 2 were 96.8 for the full CPD group (64%), 99.5 for the partial CPD group (66%) and 95.1 for the control group (63%). Again, there was no statistically significant difference in post-test scores between the different groups [*F*_2,36 _= 0.484, *p* = .620], and a similar pattern in variation in scores from 63 to 134 out of 150.

The teacher colleagues’ average assessment scores at post-test 2 were similar to those of the specialists, though showed a wider spread of performance (from 47 to 142 marks). There was no statistically significant difference in the scores between the three treatment groups [*F*_2,28_ = 0.551, *p* = .583].

To explore whether science specialists had significantly more (or less) science knowledge than their colleagues, their scores were compared with each other in each of the three treatment groups. No significant differences were found [full CPD group: *F*_1,21 _= 0.953, *p* = .340; partial CPD group: *F*_1,21 _= 0.072, *p* = .791; control group: *F*_1,21 _= 1.491, *p* = .236].

Directionally, the scores all increased, with specialists’ gains being higher among the two treatment groups than the control group. This trend was not apparent for colleagues. As the scores increased across all three groups, this may reflect a difference in content of the baseline and the post-test papers rather than any absolute improvement in teacher performance

The quantitative data revealed no statistically significant findings in subject knowledge between groups and before and after the intervention.

Interviews with teachers indicated that half the teachers in the full treatment group felt there had been some improvements in subject knowledge. For example:

… It’s been quite nice to refresh [subject knowledge] when they’ve been asking questions about various things. Sort of, lurking, I’ve been able to pull that … and then it’s, sort of, built on that knowledge, and thinking things that we weren’t quite sure … so it’s – yes, it’s answered a lot of misconceptions that perhaps we had at grammar school. … . There’s lots of things that I feel I now have stored in here [taps head]. (Science specialist, School 3, full intervention)

Participants were, however, more likely to report improvements in levels of confidence in teaching science and in executing their roles as subject leaders. This was the case for three-quarters of the participants. For example:

I’m much more confident in delivering science … and I think that’s to do with the CPD. (Science specialist, School 85, partial intervention)

… I feel a lot more confident in advising other teachers about science … (Science specialist, School 86, full intervention)

Increased confidence was also reported by teaching colleagues and senior managers, for example:

I think she is more aware of what she is doing, she’s got more direction … if I go to her now looking for help she can tell me, ‘You can do this, you can do that, you can do this type of thing.’ Whereas before it was, it took more of a discussion rather than a leadership role. (Teaching colleague, School 58, full intervention)

… we have a science specialist who is on the ball now and knows what she’s talking about and has had training … in terms of bringing the new curriculum in, I think that’s supported [her] in feeling confident. (Senior manager, School 78, partial intervention)

Changes in teaching approaches were also reported, with all participants reporting a shift towards more practical work, including open-ended inquiry. In the words of one teacher:

It’s forced me to be more practical and to feel guilty if I’m not practical (Science specialist, School 78, partial intervention)

Other skills were felt to have improved:

I suppose my questioning has changed my way of eliciting answers from children, and ways of finding out what they know, what they need to know next and how I can give that to them. (Science specialist, School 58, full intervention)

Lesson observations provided corroborating evidence of a shift towards hands-on investigative practical work.

Participants were encouraged to share what they had learned with teaching colleagues in their schools. Here it was clear that the most commonly shared activities were hands-on practical activities. There was, though, a feeling that this was less successful than had been hoped, with less change in colleagues’ practice being observed. However, there were occasions when teaching colleagues indicated they had changed their practice because of input from their science specialist:

One of the things I have learned is actually how to conduct a science experiment. So, the importance of scientific inquiry, predictions, evaluating, and that constant ‘why?’ My understanding of science now is that it’s for the children to explore and for children to be very hands-on and practical. (Teaching colleague, School 3, full intervention)

The teaching colleague attributed the change to three factors: first, having opportunities to observe their science specialist’s practice, second, having the confidence to try things out in their own lessons, and third, the science specialist providing direction and feedback on lessons.

Over the whole sample, more impact was reported from staff and pupils in schools where a teacher had experienced the full intervention.

### Organisational change

Data on organisational change in schools in the two treatment groups were gathered through the interviews with the science specialists, a teaching colleague and a senior manager.

School staff and senior managers were asked about the plans or actions that they currently had in place for science, how science featured in their school’s action plan, and what changes might be considered because of participation in the CPD intervention. The majority of teachers in the full and partial intervention schools reported that there had been no reduction in school science provision in their schools, and several school management initiatives continued to focus on science. Science specialists and teaching colleagues indicated that they felt science was a priority for senior managers in their schools, who were also supportive of participation in CPD and allocated resources and budget to science.

Senior managers were asked about the nature of the role of the science specialist in their school. Specific responsibilities most frequently mentioned were maintaining a high profile for science in the school, implementing new teaching approaches, and monitoring and evaluating the various elements of science teaching and learning. Participation in CPD was seen as a key element of the role, with the CPD that formed the focus of this study being seen as very helpful in implementing the changes in the national curriculum:

Because of this project we’re much further ahead for the new curriculum, we’ve already changed our schemes of work and so on … (Senior manager, School 85, partial intervention)

Senior managers were asked about how science is taught in their schools, and how participation in the CPD had influenced provision and practice. The science specialists and teaching colleagues were asked about the teaching methods they used in science lessons as a result of participating in the CPD.

All participants reported using methods that feature more pupil participation and less teacher control, with specific mention made of practical work, hands-on activities, partner talk and inquiry-based methods. This, in turn, appeared to be influencing whole-school approaches to science teaching. For example, one school adopted the use of ‘wow starters’ at the beginning of lessons throughout the school, and substantially increased the amount of hands-on pupil practical work.

Senior managers’ comments pointed to the science specialist having a key role in the impact the CPD had more widely in the school. A quarter of the science specialists saw the CPD as being more for personal benefit, resulting in little change in school provision. In contrast, where the science specialist proactively engaged with senior management to share the CPD outcomes, change was much more likely:

I don’t think we realised how much more we’d get from it as a school and as a staff. I think we appreciated it, it was part of [the science specialist’s] mission and CPD, but I think we have been very impressed by, you know, what he’s gleaned from that and been able to pass on. (Senior manager, School 86, full intervention)

### Participants’ use of new learning

Data on participants’ use of new learning were gathered through the interviews with science specialists, teacher colleagues, and school senior managers, supplemented by lesson observation.

All the science specialists in the full and partial intervention schools used ideas and activities from the CPD intervention in their own classes, with investigative work, ‘wow starters’, and assessment and monitoring practices most likely to be used.

The ideas and resources from the CPD programme were shared by all the science specialists in various ways in their schools and, in one-fifth of cases, in their local networks. This occurred during staff meetings and in-school training sessions. Virtually all sharing was of ideas and resources for the teaching of science, with no mention of subject knowledge. The most popular aspects shared in the full intervention schools were ‘wow starters’, investigations, practical and hands-on activities. As one science specialist said:

I did a session with all the staff on investigations. I was very keen on the one we used on the programme … I found it really helpful and I use it in class … and the children seemed really to like it. (Science specialist, School 94, Full intervention)

Uptake of the ideas and resources by teacher colleagues was modest, reported in half the full intervention schools and one of the partial intervention schools. This caused some frustration for the science specialists, as the specialist in School 94 above went on to say:

But the staff, there was just this real reluctance to use it … I felt there was a “I don’t want to do something wrong” … I think it’s a matter of confidence but also, I think, a reluctance to change. (Science specialist, School 94, Full intervention)

### Pupil learning outcomes

Pupil outcomes were assessed through subject knowledge assessments and science specialist and teaching colleague interviews.

Assessments were administered to two classes in each school, one taught by the science specialist, and one taught by their colleague. Table 6 shows the number of pupils by treatment group that completed the assessments at each stage of the project.

**Table 6.** Numbers of pupils taking assessments by treatment group.

Treatment	Baseline	Post-test 1	Baseline and post-test 1	Post-test 2	Baseline and post-test 2
Full intervention	594	486	472	491	471
Partial intervention	717	500	477	584	550
Control	669	475	475	582	561
*Total*	1,980	1,461	1,424	1,657	1,582

At baseline, 1980 pupils completed the assessments. This decreased to 1461 at post-test 1, a small number of whom had not completed the baseline assessment (14 full intervention and 23 partial intervention), leaving 1,424 for analysis. At post-test 2, the number completing the assessments in the full intervention schools remained about the same whereas for partial intervention and control the numbers returning assessments increased considerably. This allowed pupils who completed the baseline but did not complete post-test 1 to be included in the final analysis which compared baseline to post-test 2.

The maximum mark for each of the year group’s assessments differed and the scores were standardised, within each year group, by converting to z-scores allowing all the pupil scores to be combined and analysed as one group.

Table 7 shows the mean percentage scores at the three data collection points for each Year across all treatment groups. There is a consistent pattern across all three treatment groups: for pupils in Years 4 and 5 at baseline, the average mark increased when they re-took the assessment at the end of the first year, then fell or stabilised at the end of the second year because they were taking a harder assessment on the final occasion. In contrast, Year 3 averages dipped between the baseline and first post-test, and increased in the second post-test. The explanation is that this group of pupils shifted to a more difficult assessment at post-test 1 (because of potential ceiling effects with the baseline assessment) and then repeated it at post-test 2. This was not seen as an issue as it affected each treatment group equally.

**Table 7.** Pupil percentage assessment scores by year group and treatment group.

Treatment	Pupil age and year group at baseline	Number of pupils	Mean z-score (baseline)	Mean z-score (post-test 1)	Mean z-score (post-test 2)
Full intervention	7–8 (Year 3)	172	31.02	23.78	27.30
	8–9 (Year 4)	159	20.79	26.01	25.69
	9–10 (Year 5)	141	15.55	22.83	21.72
Partial intervention	7–8 (Year 3)	113	29.69	25.15	27.76
	8–9 (Year 4)	162	21.56	26.06	25.93
	9–10 (Year 5)	202	15.05	20.35	20.01
Control	7–8 (Year 3)	174	30.79	23.30	27.58
	8–9 (Year 4)	133	20.70	26.31	25.42
	9–10 (Year 5)	168	17.75	23.58	22.19

#### The baseline data

The pupil baseline data were analysed to see if there were any significant differences in assessment outcomes across the full CPD, partial CPD and control groups. The analysis revealed no statistically significant differences (*p* < 0.05) in the assessment results of the three groups: a one-way between-groups analysis of variance showed no significant differences between the groups. Raw scores, standardised z-scores and standard deviations were as follows: full CPD group: 29.6, *z* = 0.001 (*SD* = 7.24); partial CPD group: 28.3, *z* = −0.077 (*SD* = 7.12); control group: 29.67, *z* = 0.817 (*SD* = 7.45). *F*(2,736) = 2.88, *p* = 0.06.

#### The post-test 1 data

At the post-test 1 stage, after adjustment for baseline assessment score, multi-level modelling showed no statistically significant differences in post-test 1 scores between the three groups, i.e. the scores of pupils’ in the groups of full CPD were not statistically significantly higher than those for the other groups. A one-way between-groups analysis of variance showed no significant differences between treatment groups. Raw scores, standardised z-scores and standard deviations were as follows: full CPD group: 29.6, *z* = .001 (*SD* = 7.24); partial CPD group: 28.3, *z *= −0.077 (*SD* = 7.12); control group: 29.67, *z* = 0.817 (*SD* = 7.45). *F*_2,736_ = 2.88, *p* = .06.

#### The post-test 2 data

When the baseline outcomes were compared with the post-test 2 outcomes, and after adjustment for baseline assessment score, multi-level modelling again showed no statistically significant differences in post-test 2 scores among the three groups.

As there were no statistically significant differences between the pupil assessment scores of the treatment groups, effect sizes were calculated to help quantify differences between groups. After adjustment for the covariates for post-test 2 pupil scores, the effect size found for the full CPD group versus the control was 0.02, for the partial CPD group versus the control, −0.10 and for both groups combined versus the control was −0.05. The effect sizes show low or very low impact and the negative figures indicate that any difference was in favour of the control group.

In summary, the analysis indicates that the teacher CPD did not have a significant impact on pupil attainment scores.

When teachers were asked about impact on pupils, most of the responses focused on increased engagement resulting from more practical work or the use of ‘wow starters’ for lessons. On occasions, this was then linked to possible improvements in pupils’ learning:

Yeah, I think [the pupils are] engaging because they are asking the question that’s getting them to think about, well, why is that happening? It’s getting them to question and if it gets them to question and get them interested, then it follows they’ll be learning about it. (Teaching colleague, School 86, full intervention)

## Discussion

The quantitative evidence from the RCT suggests that there was little change in science teachers’ subject knowledge, and confidence regarding conceptual understanding, irrespective of whether they experienced the full or partial CPD intervention. Whilst pupils’ science knowledge increased over the period of the study, as would be expected from maturation, there was little difference between full and partial intervention schools.

The self-report data from participants points to multiple layers of learning. These included improvements in subject knowledge for science specialists in the full intervention group, change in the understanding of science as a subject, and ideas learned about how to teach science. These knowledge gains were reported as translating into affective gains such as more confidence in teaching science, increased interest in science or in teaching science, and greater confidence in handling curriculum change. In turn, these cognitive and affective gains manifested behavioural changes in the way science was taught and leadership style. Interviews with senior management staff and observation data support increased confidence, change in practice and in leadership.

The principal area where there were differences between self-report and other data was subject knowledge. Whilst more than half of the participants in the full intervention reported an improvement in their subject knowledge as a direct result of undertaking the full intervention group CPD programme this was not supported by the findings from the RCT. The RCT showed no statistically significant evidence of impact on teachers’ subject knowledge. Whilst participants might have believed that their subject knowledge had improved there was no objective evidence to substantiate such claims. One factor that may contribute to explaining this situation is participants’ perception of their subject knowledge needs. Full intervention participants had difficulty making a connection between these needs and the learning of what they felt was ‘high-level’ science content. Rather, the view was that they needed slightly more knowledge than was required to teach their pupils. Many teachers did not realise that the subject knowledge assessment used questions from the national Standard Assessment Tests for pupils aged 14, believing the demand to be higher and therefore including far more subject knowledge than necessary. Moreover, the assessments administered were wide-ranging whereas the science knowledge imparted directly by the CPD was inevitably limited.

It is important to note that external factors may have influenced the impact of the CPD. In particular, staff in schools in the partial intervention and control groups were more likely to engage in alternative science-related CPD because their school did not receive the full intervention.

## Conclusions

The study is a comparatively rare example of a mixed methods RCT combining attainment and attitude outcomes with rich case study data. It lends weight to the argument that mixed-methods approaches yield a more holistic picture of the outcomes of an intervention (Hanley et al., 2016) as they enable both the product and the process to be evaluated. In other words, they go beyond answering the question ‘does it work? (or not) to offering explanations for why this might be, and what circumstances are likely to increase chances of success. In the study reported here, a trial aimed solely at measuring the impact on pupils’ learning (Guskey’s highest level of impact) would simply have shown that the CPD intervention had not worked.

Augmenting the RCT with the interview data from the case study schools revealed that the intervention had a number of positive impacts on practice although it failed to improve pupil learning within the research timeframe. The case study data also point to some of the reasons why a measurable impact may not have been achieved. At a very basic level, the science specialist teachers struggled to persuade their teacher colleagues to adopt the ideas they had encountered on the CPD intervention. It was evident that the specialist teachers and their teacher colleagues were most attracted to practical classroom activities that they found straightforward to implement and enjoyable for pupils, with much less attention being paid to facilitating learning. Lesson observations found considerable variation in how ideas were used in the classroom as teachers adapted activities to suit their situations, raising issues about fidelity of implementation.

Teacher confidence in adopting new approaches and the ability to recognise the essential unchangeable features of an intervention are key to its success. Evidence repeatedly shows quality of teaching is a key mediator of pupil learning outcomes so interventions need to be designed accordingly. Those with a smaller number of active ingredients are often easier to implement and evaluate. Although this study showed the interpretive advantages of including case study data, recruitment to this component might have favoured schools who were particularly well-disposed towards the research and possibly towards the intervention. All these are important messages for those seeking to use CPD to bring about measurable change in classrooms and for those seeking to evaluate its success.

## Notes

1 The average attrition rate across RCTs conducted by the Education Endowment Foundation (EEF) in the UK is 15%, with projects typically having a shorter duration than two years.

2 The research team is grateful to Terry Russell and Linda McGuigan (Centre for Research in Primary Science and Technology, University of Liverpool) for permission to use assessment questions they had developed and validated.

3 The test questions for pupils aged 10–11 were drawn from national Standard Attainment Tests (SATs).
